# Combination of the Ilizarov Method and Intramedullary Fixation for the Treatment of Congenital Pseudarthrosis of the Tibia in Children: A Retrospective Observational Study

**DOI:** 10.3389/fsurg.2022.901262

**Published:** 2022-05-17

**Authors:** Ainizier Yalikun, Maimaiaili Yushan, Yimurang Hamiti, Cheng Lu, Aihemaitijiang Yusufu

**Affiliations:** Department of Microrepair and Reconstructive Surgery, The First Affiliated Hospital of Xinjiang Medical University, Urumqi, Xinjiang, China

**Keywords:** congenital pseudoarthrosis of the tibia (CPT), intramedullary fixation, ilizarov method, neurofibromatosis type 1, surgical treatment

## Abstract

**Purpose:**

Congenital pseudoarthrosis of the tibia (CPT) is a rare disease in children, and its treatment remains a challenge for orthopedic surgeons. The purpose of this study was to evaluate treatment outcomes of patients with CPT treated by using the Ilizarov method combined with intramedullary fixation.

**Method:**

Eighteen patients evaluated retrospectively from January 2009 to January 2020 were treated using the Ilizarov method combined with intramedullary fixation. Demographic data, clinical characteristics, and complications were all recorded and investigated during the period of follow-up. Ankle function was evaluated by the American Orthopaedic Foot and Ankle Society (AOFAS) ankle-hindfoot scores at the last follow-up.

**Result:**

The average follow-up was 39.2 months (25–85 months) for all 18 patients. The mean age was 6.2 years (3.5–11.2 years). Fourteen (77.8%) patients had a primary bone union at the site of pseudarthrosis, while four obtained union after secondary surgical intervention. The mean duration of the Ilizarov method was 8.1 months (4.2–13.5 months). Eight (44.4%) patients had a pin-tract infection during treatment. Four (22.2%) patients had proximal tibial valgus with a mean angle of 12.1° (5–25°), while seven (38.9%) patients had ankle valgus deformities with a mean of 10.3° (5–20°). Eleven (61.1%) patients had an average 1.4 cm of limb length discrepancy (LLD) (0.6–3.1 cm) postoperatively. Five (27.8%) patients had refracture and recovered after a secondary surgery. At the last follow-up, the average postoperative AOFAS score was 72 (55–84).

**Conclusion:**

The Ilizarov method combined with intramedullary fixation is an effective method for the treatment of CPT, which can facilitate bony union and help to prevent refracture. Management of fibular pseudarthrosis is associated with functional outcomes. It is necessary to follow up until skeletal maturity and evaluate long-term clinical outcomes.

## Introduction

Congenital pseudarthrosis of tibia (CPT) is a thorny problem in pediatric orthopedic surgery because of its unknown etiology and difficult treatment (1). Although its relationship with neurofibromatosis type 1 (NF1) was confirmed in 1950 (2), the pathophysiology of CPT remains unclear. Only 4% of children with NF-1 have CPT, while 40%–80% of children with CPT have NF-1 (3). At present, the disease is mainly treated by surgery; the purpose is to achieve bone union of pseudarthrosis, restore the normal alignment of the tibia, avoid the complications such as limb length discrepancy, ankle valgus, proximal tibial valgus and refracture, and maximize the preservation of limb function.

The Ilizarov method has been widely used in the treatment of CPT because of its high union rate (4). The advantage is that it provides mechanical stability while correcting complex lower limb deformities and bone defects, but it also carries the risk of axial offset and refracture after the removal of the fixator (5, 6). Intramedullary fixation is also effective for CPT, which can obtain bone healing and prevent refracture but is less stable for complex deformities or severe fractures of the lower extremities and can lead to stiffness of the ankle after long-term fixation (7). In recent years, some scholars have combined the advantages of these two operations in the treatment of CPT and obtained better results in achieving and maintaining bone healing and reducing residual deformities of the lower extremities (4, 8, 9). The purpose of this study is to evaluate the treatment outcome of patients with CPT treated by combining the Ilizatrov method with intramedullary fixation.

## Methods and Materials

We retrospectively analyzed 18 patients with CPT who were treated with the Ilizarov method combined with intramedullary fixation in our institution (The First Affiliated Hospital of Xinjiang Medical University) from January 2009 to January 2020. All patients were approved by our review ethics committee, signed informed consent, and received follow-up for more than 2 years. Of the 18 patients, 7 (38.9%) were males and 11 (61.1%) were females, with an average age of 6.2 years (3.5–11.2 years). All patients were involved unilaterally, 10 (52.6%) on the left and 8 (44.4%) on the right. The distal 1/3 of the tibia was affected in 16 patients and the middle 1/3 in 2 patients. All patients were classified as Crawford type IV, 9 (50%) with pseudarthrosis of the fibula and 13 (72.2%) with NF1. The average limb-length discrepancy (LLD) preoperatively was 3.2 cm (1.5–5.2 cm). Before referral to our institution, four patients underwent 1–3 operations in other hospitals but did not achieve tibial healing, and the other 14 patients had not received surgery before (Table 1).

**Table 1:** Demographic data and clinical characteristics.

Age, years	6.2 (3.5–11.2)
Gender	
Male, *n* (%)	7 (38.9%)
Female, *n* (%)	11 (61.1%)
Side	
Left, *n* (%)	10 (52.6%)
Right, *n* (%)	8 (44.4%)
Patients with previous surgery, *n* (%)	4 (22.2%)
Associated with NF1, *n* (%)	13 (72.2%)
Fibular pseudoarthrosis, *n* (%)	9 (50%)
Duration of ilizarov, months	8.1 (4.2–13.5)
Follow-up, months	39.2 (25–85)
Union	
Primary, *n* (%)	14 (77.8%)
Secondary, *n* (%)	4 (22.2%)
AOFAS score postoperatively	72 (55–84)

*AOFAS, American Orthopaedic Foot and Ankle Society.*

### Surgical Procedures

All operations in this study were performed by a surgical team. The patient was placed on an operation table in the supine position. A longitudinal incision was made at the site of the pseudarthrosis of the tibia, the periosteum of the thickened lesion at the pseudoarthrosis was incised, the pathological bone was removed, and the thickened periosteum, sclerotic bone, and surrounding fibrous tissue were further removed (the extent of excision depends on the type and length of the pathological bone). The proximal and distal medullary cavity of the tibia was opened with an electric drill, and the anterior tibial artery and posterior tibial vascular nerve bundle were fully protected during the operation.

According to the narrowest diameter of the medullary cavity, a suitable intramedullary nail or a 2.5–4 mm Kirschner wire was selected and inserted from the distal pseudarthrosis of the tibia to the epiphyseal plate of the distal tibia through the talus and calcaneus to the plantar skin. Then, the intramedullary fixation nail was hammered into the proximal medullary cavity of the tibia to the level of the tibial tubercle from the opposite direction, about 2 cm below the epiphyseal plate of the proximal tibia. After completion of intramedullary fixation, an Ilizarov external fixation ring was installed at the proximal and distal ends of tibial pseudarthrosis. After the completion of intramedullary fixation, two Ilizarov’s fixator full rings were installed at the proximal end of the pseudarthrosis according to the position of the pseudarthrosis, and 1–2 full rings were installed at the distal end of the pseudojoint and fixed with two crossed olive nails. Before fixation, proper traction of the leg was applied to correct the lower limb force line again.

Because the process of excision pseudarthrosis and correcting tibial deformity during operation will cause different degrees of bone defect, three different strategies were used for applying the Ilizarov technique. In three cases, cancellous bone graft and acute compression were applied to the bone defect. In additional three cases, the length of the bone defect was >4 cm, and the resection of the dystrophic part of the bone was responsible for tibial shortening, so segmental bone transportation was performed to avoid an LLD. Cancellous bone graft, acute compression, and lengthening were used in the remaining 12. The pathological bone and fibrous tissue of the pseudarthrosis of the fibula also need to be removed, and the fibula is fixed with a Kirschner wire. For a patient with tibial pseudarthrosis and intact fibula, the fibula osteotomy should be performed from the 3–4 cm under the fibular head to facilitate limb lengthening using the Ilizarov technique.

In 15 of the 18 patients, the ipsilateral iliac cancellous bone was grafted to the pseudarthrosis site with acute compression. In 12 cases, limb lengthening was performed simultaneously with acute compression, and appropriate pressure was applied to the tibial pseudarthrosis site while limb lengthening. In principle, the site of proximal tibial corticotomy in children is below the tibial metaphysis, as close to the cancellous bone as possible. In three cases with distal bone stock shorter than 3 cm, a calcaneal half-ring was used to increase ankle stability.

### Postoperative Management

**Figure 1:**
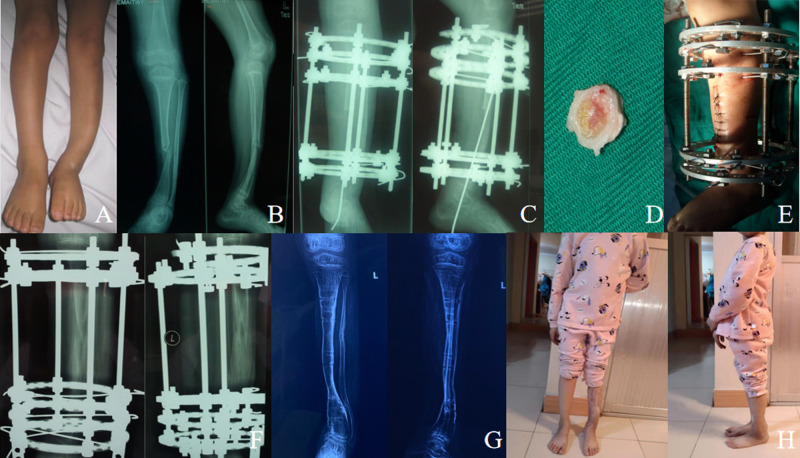
Case of a 6-year-old girl with CPT Crowford type IV associated with NF1. (**A**) Preoperative photograph showing congenital tibial pseudoarthrosis on the left side and leg length discrepancy (LLD). (**B**) Anteroposterior and lateral X-rays showing Crawford type IV CPT. (**C**) Anteroposterior and lateral radiographs viewed 2 days after resection of the pseudoarthrosis and then a Ilizarov fixator combined with intramedullary fixation of the tibia was carried out to manage the pseudarthrosis. (**D**) Excised fibrous tissue and bone of CPT. (**E**) Postoperative photos after Ilizarov fixator installation. (**F**) Anteroposterior and lateral X-ray films at 105 days after operation when docking site was reached; the intramedullary K-wire was removed because of the obvious pin-tract infection in the plantar soft tissue. (**G**) Anteroposterior and lateral radiographs taken at age 9 years showing a well-aligned tibia and bone union. (**H**) Function recovery at 41 months after removal of the external fixator.

**Figure 2:**
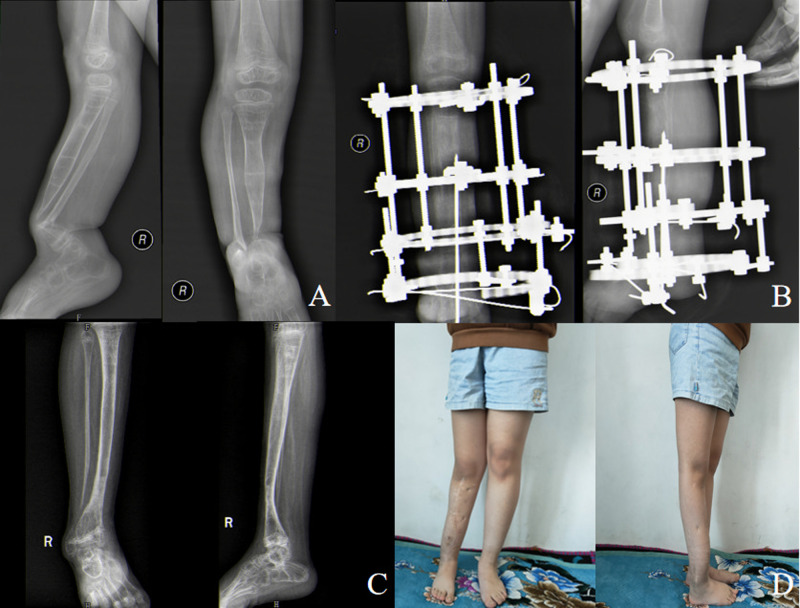
Typical case of a 5.5-year-old girl with congenital pseudarthrosis of the tibia. (**A**) Preoperative anteroposterior and lateral X-ray showing Crawford type IV CPT and intact fibula. (**B**) Anteroposterior and lateral X-ray films at 98 days after operation, showing good consolidation of distracted region. (**C**) Anteroposterior and lateral radiographs at 40 months after removal of the external fixator, showing a well-aligned tibia and bone union. Thirty-six months after the external fixator was removed, the intramedullary K-wire was removed in order to restore the range of motion of the ankle joint. (**D**) Forty-one months after removal of the external fixator, the patient’s standing and walking function recovered.

For cases with proximal tibial lengthening and internal bone transport, distraction osteogenesis was started 3–5 days after operation, and the rate and rhythm was 1 mm/day, which was completed 4–6 times. Early postoperative rehabilitation exercises such as partial weight-bearing walking with walking aids to stimulate osteogenesis, isometric contraction of lower limb muscles, and passive movement of adjacent joints are encouraged. The rate and rhythm of distraction were adjusted according to patient tolerance and radiographic evaluation of the distracted region. To prevent joint contracture, patients were followed up every 2 weeks in the first 3 months after discharge and every 4 weeks in the next 3 months, and physical and radiographic examinations were performed to assess the pin track condition, external fixator stability, and adjacent joint range of motion. When the bridging callus appeared radiographically and limb length equalization was achieved, the frame was dynamized in order to assess the mechanical stability of the regenerated bone until clinical radiographic healing was achieved. The Ilizarov fixator was removed after an X-ray taken in two planes showed corticalization of four cortices (10). The leg was protected in a long-leg cast for 12 weeks, with the patient using only partial weight-bearing. This was followed by a protective short leg orthosis until skeletal maturity (typical case in Figures 1, 2).

### Evaluation

At the last follow-up, the healing of pseudarthrosis of the tibia was evaluated. Union of congenital pseudarthrosis of the tibia was defined as bridging callus on four cortices across the transverse tibial cortical defects, with no visible fracture line on the anteroposterior and lateral radiographs (10). Complications such as pin-tract infection, ankle valgus, proximal tibial valgus, and LLD were recorded, and the AOFAS score, primary union rate, and refracture rate were assessed to evaluate the final clinical outcome.

Union was labeled as being “primary” when it united after the present surgery without any need for secondary surgeries (excluding external fixator removal and casting or change of wire, which was done under general anesthesia in some patients) and secondary when additional surgery was required to obtain union (11). A patient who had >3° of proximal tibial valgus and >5° of ankle valgus was defined as proximal tibial valgus and ankle valgus deformity, respectively (8).

## Results

A total of 18 patients were enrolled in this study, with an average follow-up of 41.2 months (25–85 months). A total of 14 (77.8%) patients with pseudarthrosis of tibia had a primary union. Of the four patients who did not achieve primary union, three cases were healed by cancellous bone graft and acute compression and one case was healed by cancellous bone graft combined with plate fixation. The mean duration of the Ilizarov method was 8.1 months (4.2–13.5 months).

There were no surgical complications in our study, such as neurovascular injury or compartment syndrome. During the follow-up, pin-tract infection occurred in 8 (44.4%) cases, which were managed by regular dressing or oral antibiotics. At the last follow-up, 7 patients (38.9%) had ankle valgus, with an average of 10.3° (5–20°), 4 patients had proximal tibial valgus, with an average of 12.1° (5–25°), and 11 patients (61.1%) had LLD, with an average of 1.4 cm (0.6–3.1 cm). There were five (27.8%) patients with refracture, two patients had refracture due to the insufficient contact cross-sectional area of the healed segment, and three patients had noncompliance with bracing. Of the five patients with refracture, two cases were healed by cancellous bone graft and acute compression and 3 cases were healed by cancellous bone graft combined with plate fixation (Table 2).

**Table 2:** Complications.

Pin-tract infection, *n* (%)	8 (44.4%)
Proximal tibial valgus, °, *n* (%)	4 (22.2%)
	12.1° (range, 5–25°)
Ankle valgus, °, *n* (%)	7 (38.9%)
	10.3° (range, 5–20°)
LLD postoperatively, cm, *n* (%)	11 (61.1%)
	1.4 (0.6–3.1)
Refracture, *n* (%)	5 (27.8%)

*LLD, limb length discrepancy.*

An angle of <5° is acceptable for patients with proximal tibial valgus, while more severe valgus deformity requires surgical correction. One case had 20° proximal tibial valgus, and her family refused surgical correction. Four patients with previous surgery in other hospitals had different degrees of ankle valgus at admission, and three of them still had ankle valgus after the end of treatment, which was 12°, 15°, and 10°, respectively. Three patients received Ilizarov’s bone transfer, one patient achieved primary union, two patients with nonunion were healed by cancellous bone graft combined with plate, and cancellous bone graft combined with acute compression. At the last follow-up, the average postoperative AOFAS score was 72 (55–84). All patients received satisfactory treatment and all tibial pseudarthrosis healed. Patients’ data are provided in Table 3.

**Table 3:** Patients’ data.

PN	Sex	Age, years	Associated with NF1	Follow-up months	Crawford classificati-on	FP	LLD pre-op, cm	ilizarov strategy	PU	Re	Secondary treatment	AV, °	PTV, °	LLD post-op, cm	AOFAS	Duration of Ilizarov, months
1	F	3.8	Y	50	IV	Y	2.6	BG,CL	N	N	BG,Compression	0	5	1.4	77	7.7
2	M	3.5	Y	44	IV	N	3.8	BG,CL	Y	N	–	5	0	1.2	65	8.9
3	F	5.4	Y	43	IV	N	2.6	BG,CL	Y	Y	BG + plate	0	0	0	72	7.5
4	F	7.7	Y	35	IV	Y	2.1	BG,Compression	Y	N	–	0	0	0	77	7.5
5	M	6.1	N	41	IV	N	3.9	BG,CL	Y	N	–	0	0	2.1	70	6.5
6	F	6.8	Y	85	IV	N	4.8	BT	N	N	BG,Compression	0	0	0	82	13.5
7	M	3.9	Y	31	IV	Y	2.1	BG,CL	Y	N	–	0	0	1	67	4.5
8	F	4.2	Y	35	IV	Y	3.9	BG,CL	Y	Y	BG,Compression	10	0	1.8	69	7.6
9	F	6.1	N	38	IV	N	2.3	BG,CL	Y	N	–	0	0	0.6	78	7.1
10	F	8	Y	25	IV	N	2.7	BG,CL	Y	N	–	0	0	1.1	77	7.5
11	F	6.8	N	30	IV	Y	2.4	BG,CL	Y	N	–	0	0	0	73	6.7
12	M	11.2	N	41	IV	Y	5.2	BT	N	Y	1.BG + plate 2.BG,Compression	25	8	3.1	55	12.6
13	F	5.5	Y	61	IV	Y	3.4	BG,CL	Y	N	–	12	20	1.3	60	9.7
14	M	6.1	Y	41	IV	N	4.2	BG,CL	N	N	BG,Compression	10	0	0	73	9.5
15	M	5.1	Y	40	IV	N	1.9	BG,Compression	Y	N	–	0	0	0	84	5.1
16	M	7.6	N	40	IV	Y	3.5	BG,CL	Y	Y	BG + plate	8	0	1	70	9.7
17	F	6.1	Y	29	IV	N	1.5	BG,Compression	Y	N	–	0	0	0	79	4.2
18	F	8.3	Y	32	IV	Y	5	BT	Y	Y	BG + plate	15	8	1.3	64	10.2

*PN, Patient number; NF1, neurofibromatosis type 1; FP, fibular pseudoarthrosis; LLD, limb length discrepancy; PU, primary union; Re, refracture; AV, ankle valgus; PTV, proximal tibial valgus; AOFAS, American Orthopaedic Foot and Ankle Society; BG, bone grafting; CL, acute compressing and lengthening; BT, bone transport*.

## Discussion

CPT is rare and has a variety of clinical manifestations, ranging from simple anterolateral tibial angulation to complete nonunion with extensive bone defects, and the incidence of CPT is currently reported between 1:140,000 and 1:250,000 (12, 13). At present, the goal of treatment is to achieve long-term bone healing of the tibia, prevent LLD, refracture and mechanical axis deviation of the tibia and nearby joint, and improve the quality of life of patients.

Since the end of the last century, Ilizarov’s technique has been widely used in the treatment of CPT. In a multicenter study carried out by the European Paediatric Orthopaedic Society (EPOS) in 2000 On 340 patients (14), the Ilizarov technique was shown to be the best surgical technique because of stable fixation with circular rings and preservation of alignment and length by the lengthening created by bone segment transport, and the CPT healing rate was reported to be 75%, with an average age of 7.5 years. Choi et al. (5) described the advantages of the Ilizarov technique in the treatment of CPT. An Ilizarov external fixator not only solves the complex deformity associated with CPT but also can be used in the treatment of refracture, bone defect and LLD, and can be combined with other surgical techniques. Shah et al. (16) emphasized that Ilizarov’s technique provides better axial, angular, and rotational stability of the pseudarthrosis, which is critical for a primary tibial union. Some literature points to the strong advantages of the Ilizarov technique in the treatment of CPT but also the lack of protection against refracture after removal of the external fixator, often associated with high refracture rates (14, 17). Horn et al. (18) proposed the Ilizarov technique in the treatment of 15 patients with CPT, 10 (66.7%) achieved primary union, however, all but one patient developed refracture within 4 years. Vanderstappen et al. (19) reported 12 patients with congenital tibial pseudarthrosis treated with the Ilizarov technique and followed up for an average of 24.5 years, with a refracture incidence of 6/12 (50%). Borzunov et al. (4) treated 28 patients with CPT with the Ilizarov technique alone and followed up for 2–9 years. The incidence of refracture was 17/28 (60.7%). Agashe et al. (11) treated 15 cases of CPT by intramedullary rodding combined with the Ilizarov technique, with an average follow-up time of 4.5 years, 14 patients achieved bone union, and the incidence of refracture was 15 (6.7%). Mathieu et al. (20) reported that intramedullary rodding combined with the Ilizarov technique was used to treat 10 patients with congenital tibial pseudarthrosis, and the incidence of refracture was 1/10 (10%) after an average follow-up of 4 years. According to the results reported in the above literature works, the incidence of refracture of congenital tibial pseudarthrosis treated with the Ilizarov fixator alone is high, while the incidence of refracture treated with intramedullary fixation combined with the Ilizarov fixator is relatively low. Most literature works unanimously recommend that intramedullary fixation combined with the Ilizarov method in the treatment of CPT to prevent refracture (4, 18, 21). This approach increases stability, preserves long-term alignment, and/or guides lengthening, and the rate of refracture is reduced in more than 50% of cases (22). El-Rosasy and colleagues also recommended that the refracture rate decreased from 68 to 29% in patients treated with both intramedullary rodding and external fixation in their study (9).

Combining the advantages of the above surgical methods, the Ilizarov fixator can provide a high union rate and good alignment control, intramedullary fixation can stabilize pseudarthrosis and avoid refracture, and autogenous cancellous bone graft can appropriately increase the healing area of pseudarthrosis after osteotomy and improve the healing rate. In our study, 18 patients received the above treatment, 14 (77.8%) patients achieved primary union at a mean follow-up of 39.2 months, another 4 patients achieved union after the secondary surgery, and 5 (27.8%) patients showed refracture, and the refracture rate was lower than those in previous literature works (4, 19). Intraoperative radical resection of tibial pseudoarthrosis and correction of the mechanical axis result in bone defects in several cases, and three patients in this study had bone defects greater than 5 cm in length measured intraoperatively, and segmental bone transportation was performed to avoid an LLD. However, some patients still had ankle valgus, proximal tibial valgus, LLD, and fracture after the end of treatment, which was similar to Choi’s (5) and Agashe’s (11) studies.

Postoperative ankle valgus is a common complication in the treatment of CPT. There were seven (38.9%) patients with ankle valgus deformity in our study, with an average of 10.3° (5–20°). Ankle valgus is thought to be associated with insufficient lateral support provided by the distal fibula; this is in accordance with Vanderstappen’s observations, which showed that two out of three patients had a lateral distal tibia angle (LDTA) of less than 80°and that both had fibular pseudoarthrosis (19). Although more than half of the 10 patients with nonunion of fibula had no ankle valgus, Johnston et al. (13) insisted that ankle valgus was related to persistent pseudarthrosis of the fibula. In their long-term follow-up study, Dobbs et al. (7) found that most ankle valgus occurred in patients with fibular pseudarthrosis, even though fibula lesions had been treated. Five of the seven patients with ankle valgus in our study had associated fibular pseudoarthrosis, and we believe that patients with fibular pseudoarthrosis have a tendency to increase ankle valgus postoperatively. According to the literature, supramalleolar distal tibia osteotomy to correct ankle valgus is considered the least attractive treatment option since recurrent pseudarthrosis may develop in up to 50% of the patients after this procedure (18). Steven et al. proposed that ankle valgus should probably be addressed by a medial hemi-epiphysiodesis at an appropriate time, a technique that is described as being effective for a variety of congenital conditions (23). Most of the patients in our study had an ankle valgus angle <13°, and all refused surgery to correct ankle valgus deformity. Proximal tibial valgus occurred in four patients (22.2%) postoperatively, with an average of 12.1° (range, 5–25°). We speculate that dysplasia of the proximal tibia may lead to unbalanced growth of the proximal tibia, which may be the cause of tibial valgus in our series, similar to the conclusion reached by Zhu et al. (8).

There were 11 patients with postoperative LLD, with an average of 1.4 (0.6–3.1) cm, of which 2 cases had shortening of 2.1 and 3.1 cm, respectively. They were treated with a heightening insole, and no surgical intervention was performed for other LLD less than 2 cm. In the past, in the treatment of CPT, the focus was on achieving bony union of the pseudarthrosis of the tibia while ignoring the treatment of the fibular pseudarthrosis. In recent years, some scholars have found that fibular pseudarthrosis affects the healing of tibial pseudarthrosis and ankle stability and is also associated with refracture. Tudisco et al. (24) stated that fibular pseudarthrosis appeared responsible for most of the poorest results. Persistent fibular pseudarthrosis was also found to be related to a failure of tibial union (25) and progressive valgus of the ankle. Pannier (22) suggested that the treatment of fibular pseudoarthrosis may be underestimated by some scholars, and persistent fibular pseudoarthrosis is indeed the cause of increased ankle valgus and refracture rates (15, 22). We found that among the five patients with refracture, four cases were associated with fibular pseudarthrosis, and we advocated that the management of the fibula pseudarthrosis should not be ignored during the treatment of tibial pseudoarthrosis. Some scholars had reported (11) that when 15 patients with CPT were treated with the Ilizarov technique and intramedullary rodding, the average AOFAS score was 64. Shah and colleagues (26) treated 38 patients with CPT and followed up for a long time, and the final AOFAS score ranged from 70 to 98 (mean, 83.3).

The risk factors for refracture after primary union remain controversial in the literature works (27, 28), with previous studies making the following recommendations: younger age at surgery, a smaller cross-sectional area at the union, tumor recurrence, persistent fibular pseudarthrosis, residual ankle valgus, removal of intramedullary rods, and noncompliance with the brace (5, 29). Cho (28) believed that the risk of refracture is significantly determined by atrophic congenital pseudarthrosis of tibia, age of surgery, cross-sectional area of the tibial union, and stability of fibula. Some scholars (11) considered that there are only three important causes of refracture, that is, (1) axial deviation leads to increased stress and fracture, (2) loss of intramedullary support after intramedullary fixation removal, and (3) noncompliance with the use of bracing after removal of the external fixator. Few studies have tried to determine the most effective, safe, and practical treatment strategies to minimize the residual challenges in the treatment of CPT until Choi et al. recommended the creation of a cross-union between the tibia and fibula for CPT cases where the fibula was broken but minimally proximally migrated. They converged the two fibula bone ends toward the two tibia bone ends in what they called a “4-in-1 Osteosynthesis” (29), and Paley et al. (6, 30) recently reported the results of 17 CPT treated using the Paley cross-union protocol using an external fixator. Preliminary results with intentional tibiofibular cross-union from Choi et al. (29) and Paley (30) report a probability of primary union without refracture of 100%.

It is generally accepted that pathological periosteum is one of the key factors in the etiology of CPT. Pathological periosteum is embedded and hinders the osseous connection and is also the cause of bone angiogenesis disorder (31, 32). Pathological periosteum can form fibrous bands, which increase the local pressure on the surrounding bone. This in turn leads to angiogenesis disorders and bone atrophy, and the decrease in periosteal blood supply may affect bone healing. Carlier et al. (33) emphasized lesions at the pseudoarthrosis site surrounded by abnormal hyperplastic periosteum, which may result in the limitation of angiogenesis and affect bone healing. In conclusion, vascular abnormalities at the pseudarthrosis site may be one of the important factors in the formation of pseudarthrosis. Hermanns-Sachweh et al. (31) suggested the thickened periosteum was caused by neural-like cells that form a tight sheath around the small periosteal vessels, causing narrowing or obliteration of the vessels. This results in a disturbance of the blood circulation of the periosteum, which in turn results in impaired oxygen and nutrient supply of the subperiosteal bone with subsequent fracture and recalcitrant nonunion (31, 34). Kuorilehto et al. (35) found that the arteriovenous wall thickening, lumen stenosis and vascular wall thickening, and partial obstruction of the vessels were observed. The findings of the above scholars are sufficient to provide a basis for extensive resection of pseudarthrosis and its thickened periosteum and fibrous tissue.

The limitations of this study include its single-center retrospective design and a relatively small case number to reach a strong conclusion because CPT is particularly rare. The average follow-up in our study was 39.2 months (25–85 months), and CPT has a higher risk of refracture after bone union, so it is more important to follow up to bone maturity and evaluate long-term clinical outcomes.

## Conclusion

The Ilizarov method combined with intramedullary fixation is an effective method for the treatment of CPT, which can promote bone healing and reduce the rate of refracture. Determining the state of the fibula could be very helpful in the CPT prognosis. It is necessary to follow up until skeletal maturity and evaluate long-term clinical outcomes.

## Data Availability

The raw data supporting the conclusions of this article will be made available by the authors, without undue reservation.
